# Nicolau Syndrome after Intramuscular Benzathine Penicillin Injection

**Published:** 2014-11

**Authors:** Morteza Noaparast, Rasoul Mirsharifi, Fezzeh Elyasinia, Reza Parsaei, Hessam Kondori, Sara Farifteh

**Affiliations:** Department of General Surgery, Imam Khomeini Hospital, Tehran University of Medical Sciences, Tehran, Iran

**Keywords:** Syndrome, Benzathine penicillin, Injection

## Abstract

A 3-year-old boy was admitted to the emergency department with right lower limb pain, edema, and livedoid discoloration that occurred immediately after intramuscular injection of benzathine penicillin. The patient was diagnosed with Nicolau syndrome, a rare complication of intramuscular injection presumed to be related to the inadvertent intravascular injection. It was first reported following intramuscular injection of bismuth salt, but it can occur as a complication of various other drugs. Fasciotomy was carried out due to the resultant compartment syndrome and medical therapy with heparin, corticosteroid, and pentoxifyllin was initiated.

## Introduction

Nicolau syndrome (livedoid dermatitis) is a rare complication of intramuscular injection. It was first reported following intramuscular injection of bismuth salt, but can occur as a complication of various other drugs such as nonsteroid anti-inflammatory drugs, corticosteroids, local anesthetics and interferon alpha. It manifests itself by pain, edema and livedoid skin lesions at the site of injection. Most cases cure without significant complications; however, this is not always the case. While skin necrosis is a common consequence that may require skin graft or heal with an atrophic scar, but limb ischemia may occur.^1^^-^^3^ De Sousa et al. has reported a death following Nicolau syndrome.^4^ In this report, we describe a 3-year-old boy with a diagnosis of the Nicolau syndrome after intramuscular benzathine penicillin injection.

## Case Report

**Figure 1:**
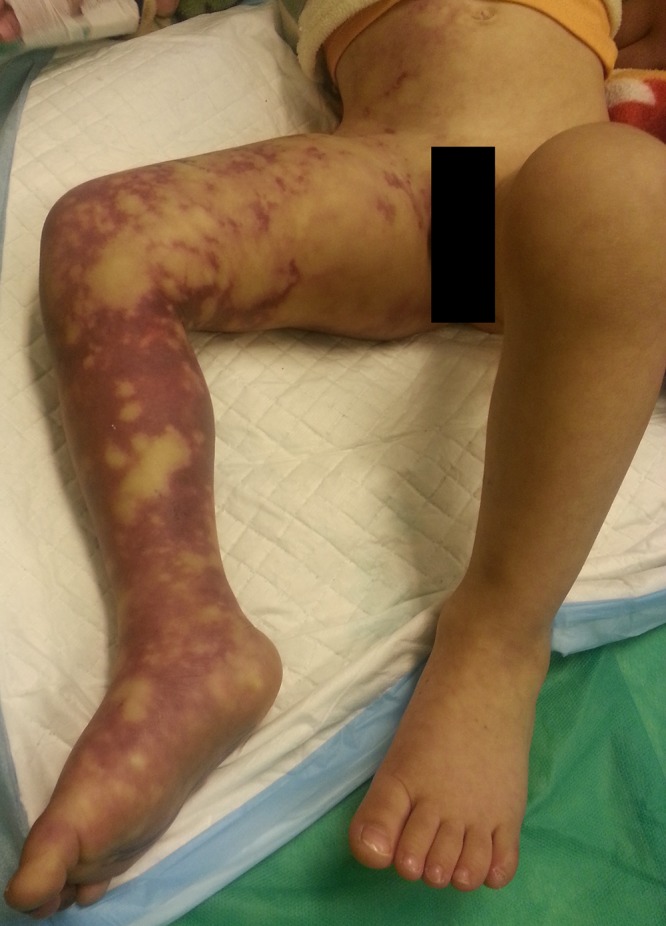
Livedoid discoloration and tense edema in patient’s lower limb.

Note: The photo in figure 1 was taken in the presence and with the permission of patient’s parent for the sole purpose of scientific publications without disclosing his identity.

A 3-year-old boy was admitted to the emergency department with swelling and skin lesions on the right lower limb. The patient had symptoms of upper respiratory tract infection for a few days prior to admission to an outpatient clinic. He received intramuscular benzathine penicillin in the upper outer quadrant of the right buttock a day before being admitted to our center. The patient developed pain, swelling, and skin discoloration immediately after the injection and was referred to our center 24 hours later. Initial examination revealed that the patient is not critically ill, as his vital signs were stable. Marked edema and livedoid erythematous discoloration was noted on the right lower limb and the lower abdomen (figure 1). The right calf was cold, tense, and popliteal, dorsalis pedis and posterior tibial pulses were absent. Electrolytes, BUN, Cr, white cell count and hemostatic tests were within the normal range. Color Doppler sonography was performed and no flow distal to popliteal artery was detected. The patient was taken to an operating room and fasciotomy of the calf was performed. Intravenous heparin, pentoxifyllin and systemic corticosteroids were initiated. Patient’s limb became warm, capillary filling was normal and his condition gradually improved. Finally, the patient’s wound was closed and he was discharged ten days after fasciotomy.

## Discussion

Nicolau syndrome (also known as livedoid dermatitis) is a rare complication of intramuscular injection, which is manifested by pain, edema, and livedoid discoloration of the skin immediately after injection. It was first described in 1925 by Nicolau following intramuscular injection of bismuth salt, but it also has been reported after intramuscular or subcutaneous injection of numerous drugs.^1^^-^^3^

Nicolau syndrome involves the skin, subcutaneous, and muscle tissue with possible necrosis. Its pathogenesis is not well understood, but few causes have been postulated. Inadvertent intravascular injection and the resultant emboli of crystals or intimal damage of the vascular tree or nerve injury with consequent vasospasm may cause this syndrome.^2^ Immunologic nature for Nicolau syndrome is ruled out since it is not observed after subsequent injection of the same drug.^1^ There are reports that subcutaneous injection instead of intramuscular injection is a predisposing factor. Okan and Canter described subcutaneous injection and injury to cutaneous arteries as a probable cause leading to subcutaneous fat and skin necrosis.^3^ However; this was unlikely in our patient since there is unsubstantial subcutaneous fat in children that makes intramuscular injection difficult. Various degrees of skin or muscle necrosis is observed and as in the case of our patient, soft tissue edema and inflammation may lead to compartment syndrome and warrant fasciotomy to prevent irreversible ischemia.^4^^,^^5^

Diagnosis is based on clinical findings since biopsy and histological examination yieldsnon-specific necrosis and inflammation.^6^ Typical features immediately after intramuscular injection are; pain, edema and livedoid erythematous or violaceous skin discoloration. Nicolau syndrome may be similar to vasculitis or cholesterol embolia. The fact that nicolau syndrome occurs immediately after injection; it is differentiated from vasculitis and distribution of skin lesions in the site of injection. This is in contrast with cholesterol embolia that involves toes and distal limb. Subcutaneous injections of drugs, intended for intramuscularly injection, are considered a risk factor for complications similar to Nicolau syndrome.^3^ Burbridge measured gluteal subcutaneous fat from 298 patients by CT-scan and found that a 37 mm needle would not penetrate the gluteal muscle fibres in 81 of 148 female patients (54.7%), in 21 of 150 male patients (14%), and in 102 of the 298 total sample (34.2%).^7^

According to findings of Dietrich et al., it is suggested that in the upper outer quadrant of the buttock, a 90 kg patient requires a 2 inch needle and a 45 kg patient requires 1.25 inch to 1.45 inch needle.^8^ Thus, if uncertainty exists regarding the adequacy of needle size, an alternative injection site should be considered.^7^

Due to the low incidence of this complication and the absence of trials, a standard treatment with proven benefit cannot be referenced, but supportive treatments like anticoagulation, pentoxyphylin, hyperbaric oxygen and steroids have been attempted.^9^^,^^10^ When Nicolau syndrome is suspected, it is prudent to avoid cold compress because of its potential to induce vasoconstriction and deterioration of ischemia. Senel et al. reported cold compress as an aggravating factor in their Nicolau syndrome patient.^11^ Patients must be closely monitored for possible limb ischemia similar to the present case where fasciotomy may become necessary. Antibiotics are used when an infection is present and surgical debridement and fasciotomy is performed if necessary. Most patients eventually heal, but atrophic scars are common.^12^ Sometimes, complete necrosis of skin occur that mandates skin graft. More sever presentations leading to limb loss or even death has been reported.^3^^,^^13^^,^^14^

In early treatment phase of this patient, a flaw in the quality of the used medicine was alleged as the cause of such complication. According to manufacturer’s feedback based on the product batch number and our literature survey, such hypothesis proved to be incorrect. Currently there is considerable concern in the public and the media regarding the quality of medicines and medical equipment. It makes diligent study and follow-up of complications necessary to find the true causes and to offer appropriate preventions. Adherence to proper injection techniques can minimize complications. The injection should be applied in the upper outer quadrant of the buttock and aspirating the needle before injecting the medication to ensure that no inadvertent intra-arterial injection occurs. 

## Conclusion

Physicians must be aware of possible complications of intramuscular injection and particularly avoid unnecessary injection in children. Resistance to antibiotics is a probable consequence of antibiotics overuse. Anaphylaxis is a feared complication of penicillin injection, but complications of intramuscular injection are not limited to these widely recognized problems. In the case of our patient, we could not judge the decision for prescribing penicillin; however, we know that the overuse of antibiotics continues to haunt the health care system. Encountering such cases, further underscores the importance of rational prescribing of medicines. 

## 

**Conflict of Interest:** None declared.
